# Comparative Study on Antioxidant Capacity of Diverse Food Matrices: Applicability, Suitability and Inter-Correlation of Multiple Assays to Assess Polyphenol and Antioxidant Status

**DOI:** 10.3390/antiox14030317

**Published:** 2025-03-06

**Authors:** Attila Kiss, Vivien Anna Papp, Anna Pál, József Prokisch, Sara Mirani, Bela E. Toth, Tarek Alshaal

**Affiliations:** 1 Agro- and Food-Industrial Research Centre, Széchenyi István University, Egyetem Sqr. 1, 9026 Győr, Hungary; 2 Centre for Agro- and Food-industrial Innovation, Faculty for Agro and Food Sciences, University of Debrecen, Böszörményi Str. 138, 4032 Debrecen, Hungary; papp.vivien@agr.unideb.hu (V.A.P.); pal.anna@agr.unideb.hu (A.P.); 3 Department of Animal Husbandry, Faculty for Agro and Food Sciences, University of Debrecen, Böszörményi Str. 138, 4032 Debrecen, Hungary; jprokisch@agr.unideb.hu; 4 Department of Pharmaceutical Surveillance and Economics, Faculty of Pharmacy, University of Debrecen, Egyetem Sqr. 1, 4032 Debrecen, Hungary; sara.mirani@pharm.unideb.hu (S.M.); tothe.bela@pharm.unideb.hu (B.E.T.); 5 Department of Applied Plant Biology, Faculty of Agricultural and Food Sciences and Environmental Management, University of Debrecen, Böszörményi Str. 138, 4032 Debrecen, Hungary; 6 Soil and Water Department, Faculty of Agriculture, University of Kafrelsheikh, Kafr El-Sheikh 33516, Egypt

**Keywords:** bioactive compounds, radical scavenging activity, reducing power, nutritional profiling, health-promoting properties, comparative bioactivity

## Abstract

Antioxidants play a crucial role in mitigating oxidative stress and preventing cellular damage caused by free radicals. This study aimed to compare the effectiveness of three antioxidant assays—DPPH, TEAC, and FRAP—in quantifying the antioxidant capacity of 15 plant-based spices, herbs, and food materials from five distinct plant families. The relationship between these assays and total polyphenol content (TPC) as well as total flavonoid content (TFC) was also investigated. The results showed that FRAP exhibited the strongest correlation with TPC (r = 0.913), followed by TEAC (r = 0.856) and DPPH (r = 0.772). Lamiaceae species, such as rosemary and thyme, consistently demonstrated high antioxidant activities across all assays. The study highlights the complementary nature of these assays in assessing antioxidant capacity and underscores their utility in profiling polyphenol- and flavonoid-rich plants for potential nutritional and therapeutic applications.

## 1. Introduction

Antioxidants constitute a group of molecules that, thanks to their stable structures, can neutralize free radicals by electron transfer, as well as H-donor capability, thus reducing their harmful impacts. They delay or inhibit cell damage by their so-called free radical scavenging properties [1]. Antioxidants play an important role in protecting substances in living cells against oxidation [2]. Numerous studies have shown that the increased intake of plant-based antioxidant compounds exerts several beneficial effects on the health of consumers and contributes to enhanced resistance to various diseases [3,4,5,6].

As great distinctions might be observed between the antioxidant activities of diverse plant species, it is of immense interest to make authentic comparisons of the biological relevance (antioxidant capacity) of the most commonly applied plant-based food materials. In this comparative study, 15 representatives of five distinct, widely applied plant families (Lamiaceae, Amaranthaceae, Solanaceae, Zingiberaceae, and Asteraceae families) were selected.

Lamiaceae is one of the most widely used and researched plant families, due to the various phytochemicals its species possess. These compounds have many beneficial effects on the human body, including antioxidant, antibacterial, and antitumor impacts. This family includes a large number of species, with the most common representatives, used as spices and medical herbs, being rosemary, thyme, oregano, and basil [7]. The most important bioactive compounds in this family of plants are volatile terpenoids, essential oils, hydroxycinnamic acids, and various phenolic compounds. Phenolic compounds of significant antioxidant activity are secondary metabolic products, including ferulic acid, caffeic acid, and rosmarinic acid [8,9]. Amaranthaceae is one of the most diversified plant families with varieties including beetroot, amaranth, and spinach. Plants belonging to this family have been shown to have antioxidant, anti-inflammatory, antimutagenic, antitumor, and chemopreventive properties [10]. The most important phytochemicals that occur in large quantities in plants belonging to this family include coloring agents (e.g., carotenoids), phenolic compounds, flavonoids, tannins, and glycosides as well as a number of other biologically active components with beneficial effects [11].

The most important representatives of the Solanaceae family include potatoes, tomatoes, and various types of peppers. Studies have demonstrated their versatile physiological benefits, with special regard to anticancer, antioxidant, and anti-inflammatory activities as well as blood pressure- and cholesterol-lowering impacts [12,13]. The most commonly found compounds in these plants are coloring agents (e.g., carotenoids), alkaloids, saponins, flavonoids, glycosides, and phenolic components (caffeic acid, capsinoids, etc.) [12,14]. The Zingiberaceae plant family’s best-known representatives are ginger and turmeric. They have many compounds that are beneficial to human health [15].

Asteraceae is the family with the largest number of species and the widest variety of plants, such as yarrow tail. Some of these species also have medicinal properties, associated with their bioactive components with beneficial effects [16].

Polyphenols and flavonoids are crucial bioactive compounds found in plant tissues, being responsible for their potent antioxidant properties [17,18,19]. Polyphenols, including phenolic acids and tannins, play a key role in neutralizing free radicals and reducing oxidative stress in cells, which helps to prevent damage to lipids, proteins, and DNA [20]. Flavonoids, a subgroup of polyphenols, are particularly effective at scavenging reactive oxygen species (ROS) and chelating metal ions. These compounds contribute to plants’ defense mechanisms and have been linked to human health benefits, such as reducing the risk of chronic diseases like cardiovascular disease and cancer. Their antioxidant activity is vital to maintain cellular health and mitigate oxidative damage in both plants and animals [21].

The 15 plant species studied in this research are known for their rich content of bioactive compounds, particularly polyphenols and flavonoids, which contribute significantly to their antioxidant properties. For instance, rosemary, a member of the Lamiaceae family, contains high levels of phenolic acids such as rosmarinic acid (up to 12.5 mg/g) and carnosic acid (up to 8.2 mg/g), which have been shown to exhibit strong antioxidant, anti-inflammatory, and neuroprotective effects in both in vitro and in vivo studies [22,23]. Similarly, thyme is rich in thymol and carvacrol, with concentrations ranging from 2.5 to 5.0 mg/g, and has demonstrated antimicrobial and antioxidant activities in human clinical trials [24,25]. Oregano contains a variety of phenolic compounds, including rosmarinic acid (up to 10.8 mg/g) and quercetin (up to 3.2 mg/g), which have been linked to anticancer and anti-inflammatory effects in animal models [26]. Turmeric, a member of the Zingiberaceae family, is well known for its high curcumin content (up to 3.5 mg/g), which has been extensively studied for its antioxidant, anti-inflammatory, and anticancer properties in human trials [27]. Basil contains significant amounts of rosmarinic acid (up to 6.7 mg/g) and apigenin (up to 2.1 mg/g), which have been shown to possess antidiabetic and anti-inflammatory effects in vivo [28]. Beetroot, from the Amaranthaceae family, is rich in betalains (up to 1.2 mg/g) and phenolic acids, which have been associated with antioxidant and antihypertensive effects in human studies [29,30]. Spinach contains high levels of flavonoids such as patuletin (up to 1.8 mg/g) and spinacetin (up to 1.5 mg/g), which have demonstrated antioxidant and anticancer activities in vitro [31]. Onion is rich in quercetin (up to 1.3 mg/g) and anthocyanins, which have been shown to have immunomodulatory and anticancer effects in human studies [32,33]. Tomato contains lycopene (up to 0.5 mg/g) and chlorogenic acid (up to 1.0 mg/g), which have been linked to cardioprotective and antioxidant effects in clinical trials [34]. Chili pepper is rich in capsaicinoids (up to 1.0 mg/g) and flavonoids, which have demonstrated anti-obesity and anti-inflammatory effects in vivo [35,36]. Yarrow contains apigenin (up to 1.5 mg/g) and luteolin (up to 1.2 mg/g), which have been shown to possess antioxidant and anti-inflammatory properties in animal models [37,38]. Walnut is rich in chlorogenic acid (up to 1.8 mg/g) and flavonoids, which have been associated with neuroprotective and cholesterol-lowering effects in human studies [39]. Brewer’s yeast contains phenolic acids such as gallic acid (up to 0.5 mg/g) and catechin (up to 0.3 mg/g), which have demonstrated antioxidant and antitumorigenic effects in vitro [40,41]. These studies highlight the diverse range of antioxidant compounds present in the studied species and their potential health benefits, underscoring the importance of further research into their biological activities and therapeutic applications.

The studied plants, selected from various families, exhibit diverse traits in terms of tissue type and antioxidant profile, leading to considerable variations in the observed antioxidant capacities. Based on previous literature findings, predictions might be made on the approximate order of antioxidant capacity of the studied plants, which does not allow specific conclusions to be drawn on specific and intrinsic antioxidant features or direct correlations between individual components and the established antioxidant activities [39,40,41,42] therefore, it might be claimed that rosemary, thyme, and oregano have the highest antioxidant capacity based on the literature, a statement confirmed by our study.

The quantification of antioxidant capacity in plant samples is critical for understanding the potential health benefits and protective roles of bioactive compounds, such as polyphenols and flavonoids. The assessment of antioxidant activity helps characterize the plant’s ability to neutralize harmful free radicals, contributing to the prevention of oxidative stress-related diseases. The most widely used techniques include DPPH (2,2-diphenyl-1-picrylhydrazyl assay), FRAP (Ferric-Reducing Antioxidant Power), and TEAC (Trolox Equivalent Antioxidant Capacity), whose main action mechanism is principally considered to be SET (Single Electron Transfer) [43,44]. Also, the ORAC (Oxygen Radical Absorbance Capacity) assay [45], chromatographic characterization of compounds [46,47], and cellular tests [48] are of great importance.

The DPPH assay is a fast and simple method that can be applied to both solid and liquid samples. Its ease of reproducibility allows for large-scale automation, making it a highly efficient technique. Its major limitation, however, is that it does not target specific antioxidant components, which can be seen as a drawback. Additionally, the method cannot be used for measuring plasma antioxidants due to the plausible protein precipitation. Some antioxidants may react slowly or not at all with DPPH. Moreover, since DPPH is a light-sensitive molecule, the assay must be conducted in the dark to avoid degradation [44,49,50].

The primary advantage of the FRAP assay is its simplicity; it is quick, cost-effective, and does not require specialized tools or equipment. It can be used to measure the antioxidant capacity of a wide range of samples, including human, animal, and plant materials. However, its broad applicability signifies a limitation as well, as it lacks specificity in targeting particular antioxidants [50].

One of the advantages of the TEAC assay is that the radical used (ABTS: 2,2′-azino-bis(3-ethylbenzothiazoline-6-sulfonic acid)) is soluble in both aqueous and organic solvents, allowing the detection of the activity of both hydrophilic and lipophilic antioxidants. It is also applicable to both synthetic and natural antioxidants. However, some researchers question the relevance of this method, as ABTS is an artificial radical not found in biological systems. Another drawback is the longer reaction time required for the assay, which may result in an underestimation of the antioxidant capacity. Additionally, the prepared mixtures must be stored in the dark during the process [44,51,52].

It is undisputable that the complex realistic and authentic characterization of a biological sample needs the implication of diverse antioxidant assays; thus, five distinct assays were applied throughout the studies. However, we should make a distinction between the five different assays, as their action mechanisms exhibit significant differences. TPC and TFC are specific tools to assess compounds belonging to groups of either polyphenols or flavonoids, regardless of their biological function (antioxidant activity). DPPH, FRAP, and TEAC are antioxidant capacity assays, signifying that they are capable of the analysis of biologically active molecules of definite antioxidant features. As a result, the major focus point of the research was revealing the correlations of TPC and TFC with the assays of tight relation with the content of biologically active molecules (DPPH, FRAP, and TEAC). The correlation of TFC with the antioxidant assays might be affected by the fact that the DPPH, FRAP, and TEAC approaches are not necessarily focused on just flavonoid-type polyphenols, as they may be even more sensitive to a broad spectrum of non-flavonoid-like polyphenols and other reducing agents. Comparative studies have been carried out before by means of other antioxidant assays [53,54,55]; however, most of them were based on surveying the correlation of antioxidant assays by involving just a single plant. In the present paper, several plant species from different families are studied; thus, a wider range of compounds of prospective antioxidant activity were involved in the research, making possible to draw conclusions on plausible correlations between different antioxidant assays on a more comprehensive and profound basis. It signifies a significant added value to the previous research findings.

The primary objectives of this research were to compare the antioxidant capacities of plant-based substances from various families, assess the variation in the obtained data within families using different techniques, and explore the potential correlations between the applied methods.

## 2. Materials and Methods

### 2.1. Plant Materials

Fifteen well-known spices/herbs with potentially high antioxidant capacity were purchased from local Hungarian stores. The classification, antioxidant compounds, and biological importance of the selected plants are presented in Table 1. Additional pieces of information about the origin, plant part used, and the form of the spice are summarized in Table 2. The experimental design applied was the Comparative Experimental Design.

### 2.2. Source of Chemicals

Folin–Ciocalteu reagent (FCR) was purchased from Chem-Lab NV, B-8210 Zedelgem, Belgium. Gallic acid, sodium carbonate, methanol (99.8%), and catechin (trans-3,3,4,5,7-Pentahydroxyflavane) were purchased from Sigma-Aldrich, St. Louis, MO, USA. Sodium nitrite, aluminum chloride, sodium hydroxide, 2,2-diphenyl-1-picrylhydrazyl (DPPH), 6-hydroxy-2,5,7,8-tetramethylchroman-2-carboxylic acid (Trolox), 2,2′-Azino-bis (3-ethylbenzothiazoline-6-sulfonic acid) (ABTS), potassium peroxodisulfate, and 2,4,6-tripyridyl-s-triazine (TPTZ) were purchased from Thermo Fisher Scientific, China. Acetone (acetate buffer), ascorbic acid, ferric chloride, and ferric chloride hexahydrate (Fe Cl_3_ · 6H_2_O) were purchased from Sigma-Aldrich, St. Louis, MO, USA.

### 2.3. Determination of Total Polyphenol and Flavonoid Contents

Extracts were prepared using 70% methanol solution in a 1:10 sample/methanol ratio. After placing the samples in an ultrasonic bath for 30 min, they were centrifuged at 13,000 rpm for 3 min at 4 °C to obtain the supernatants. All measurements were performed in 4 replicates.

The total phenolic content (TPC) was determined in the spice/herbs extracts using the Folin–Ciocalteu reagent, following the method outlined by Singleton and Rossi [57], with absorbance measured at 760 nm. Gallic acid (GAE) was used to create the standard curve, and TPC concentrations were reported as mg GAE/g DW (dry weight). The total flavonoid content (TFC) was measured using a 10% aluminum–chloride solution, based on the protocol by [58]. The resulting absorbance was recorded at 415 nm. Rutin (CE) was utilized to generate the standard curve for TFC quantification, with concentrations expressed as mg CE/g DW. Absorbance was measured using the model UV-160A spectrophotometer (Shimadzu, Kyoto, Japan).

### 2.4. Determination of Antioxidant Capacity

Extracts were prepared using concentrated methanol solution in a 1:10 sample/methanol ratio. After incubation at room temperature in the dark for 10 min, the samples were centrifuged at 13,000 rpm for 3 min at 4 °C to obtain the supernatants. All measurements were performed with 4 repeats.

#### 2.4.1. DPPH Method

The antioxidant capacity of the studied spice/herb extracts was also determined by means of DPPH (2,2-diphenyl-1-picrylhydrazyl) radical scavenging method (Blois, 1958). DPPH reagent was prepared by dissolving 9 mg DPPH in 100 mL methanol (0.23 mM). Then, 1 mL extract was added to 2 mL of DPPH reagent, and then the sample was stored at room temperature in a dark place for 30 min. The absorbance was recorded at 517 nm using the model UV-160A spectrophotometer (Shimadzu, Kyoto, Japan). DPPH reagent served as the blank sample. The standard curve was generated using Trolox solution (10 mg Trolox dissolved in 10 mL methanol), and the concentration was expressed as mg Trolox/g DW.

#### 2.4.2. TEAC Protocol

The antioxidant activities of the selected herbs and spices were quantified with the application of the Trolox Equivalent Antioxidant Capacity (TEAC) assay, with extracts prepared as detailed in Section 2.3. Following the protocol outlined by Sharma and Singh [59], the reagent was prepared by mixing 7 mM ABTS with 5 mM potassium persulfate (K_2_S_2_O_8_) in a 1:1 ratio, followed by incubation in the dark for 12 h to allow the formation of the ABTS^•+^ radical cation. This solution was then diluted tenfold before use. The Trolox stock solution was prepared as described for the DPPH method. The reaction mixture was prepared by combining 900 µL of the diluted ABTS solution, 1000 µL of distilled water, and 100 µL of the extract. In the blank sample, 80% methanol was used in replacement of the extract. The reaction mixture was incubated under dark conditions for 20 min at room temperature. Absorbance was measured at 734 nm using the model UV-160A spectrophotometer (Shimadzu, Kyoto, Japan). The antioxidant capacity was expressed as mg Trolox/g DW.

#### 2.4.3. FRAP Assay

The antioxidant activities of the selected herbs and spices were quantified utilizing the Ferric-Reducing Antioxidant Power (FRAP) assay, with extracts prepared according to the method described in Section 2.3. Following the protocol established by Benzie and Strain [4], the FRAP reagent was formulated by combining 25 mL of sodium acetate buffer (pH 3.6) with 10 mL of 20 mM FeCl₃•6H_2_O and 10 mL of 10 mM 2,4,6-tripyridyl-s-triazine (TPZT). For the assay, 10 µL of the extract was mixed with 65 µL of distilled water and 2250 µL of the FRAP reagent. Distilled water was used in place of the extract as a blank. The mixture was incubated at room temperature for 8 min; subsequently, the absorbance was recorded at 593 nm using the model UV-160A spectrophotometer (Shimadzu, Kyoto, Japan). A 5.67 mM ascorbic acid solution was employed as the standard to construct the calibration curve. The results were reported as mg ascorbic acid/g DW.

### 2.5. Statistical Evaluation

All measurements were conducted in triplicate, except for the total phenol and flavonoid measurements, which were carried out in four replicates. The results were expressed as the mean ± SD (standard deviation). The dendrograms were performed by means of the nearest neighbor algorithm using the IBM SPSS Statistics V25.0 software. The correlation coefficients were calculated with Pearson’s test using the R statistical software. Statistical significance was established at *p* ≤ 0.05.

## 3. Results

### 3.1. Total Polyphenol and Flavonoids Contents in Spices, Herbs, and Yeast

**Figure 1:**
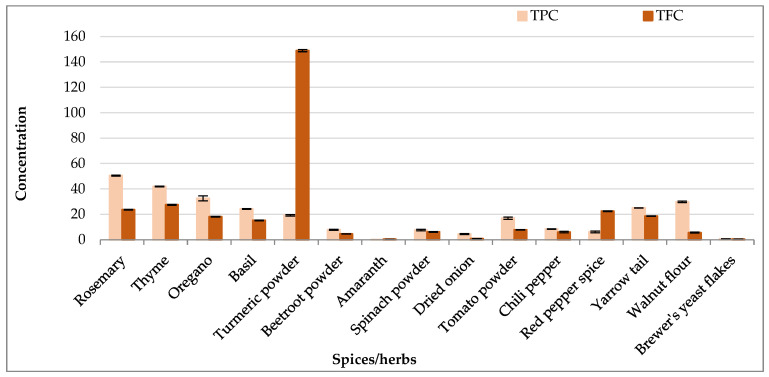
Contents of total polyphenols (TPC; mg GAE/g DW) and flavonoids (TFC; mg CE/g DW) in different plant species (spices and herbs). Data are means ± SD.

Although rosemary, thyme, oregano, and basil belong to the same botanical family (Lamiaceae) and the same plant tissues were analyzed, they exhibited significant differences in TPC, as shown in Figure 1. Rosemary displayed the highest TPC value (50.51 mg GAE/g DW), followed by thyme (41.91 mg GAE/g DW) and oregano (32.62 mg GAE/g DW), whereas basil had the lowest TPC among the Lamiaceae species (24.24 mg GAE/g DW). In contrast, the three tested species from the Amaranthaceae family—beetroot, amaranth, and spinach—showed comparatively low TPC values. Notably, beetroot and spinach powders had almost identical TPCs despite being derived from different tissues, while amaranth displayed the lowest TPC value of 0.02 mg GAE/g DW. The Solanaceae family members (tomato, chili pepper, and red pepper) displayed varying TPCs: tomato fruit powder exhibited a TPC of 16.95 mg GAE/g DW, chili pepper 8.35 mg GAE/g DW, and red pepper fruit powder 6.17 mg GAE/g DW, respectively. This variation may be explained by the use of different plant parts within these species. Walnut powder (Juglandaceae) and yarrow tail (Asteraceae) exhibited relatively high TPC values, recording 29.84 mg GAE/g DW and 25.09 mg GAE/g DW, respectively. Brewer’s yeast flakes showed a notably low TPC of 0.74 mg GAE/g DW, ranking just above amaranth. Overall, the spices and herbs from the Lamiaceae family exhibited the highest TPCs, followed by those from the Solanaceae family, while species from the Amaranthaceae family had the lowest TPC values.

The TFC reestablished for the 15 studied samples revealed substantial variations depending on the species, plant families, and tissues analyzed. Turmeric powder exhibited an exceptionally high TFC of 148.94 mg CE/g DW, likely due to the presence of strong yellow pigments, which may have affected the aluminum chloride assay. Among the members of Lamiaceae family, thyme showed the highest TFC value (27.55 mg CE/g DW), followed by rosemary (23.69 mg CE/g DW), oregano (18.12 mg CE/g DW), and basil (15.26 mg CE/g DW). In contrast, species from the Amaranthaceae family, including beetroot (4.61 mg CE/g DW), spinach (6.13 mg CE/g DW), and amaranth (0.42 mg CE/g DW), exhibited significantly lower TFCs. Solanaceae species such as tomato (7.83 mg CE/g DW), chili pepper (6.01 mg CE/g DW), and red pepper spice (22.48 mg CE/g DW) displayed moderate to high flavonoid levels. Other species, such as yarrow tail (18.64 mg CE/g DW) from the Asteraceae family and walnut flour (5.61 mg CE/g DW) from the Juglandaceae family, demonstrated intermediate TFC values. Brewer’s yeast flakes had the lowest TFC (0.42 mg CE/g DW). Overall, Lamiaceae species exhibited relatively higher flavonoid content, while Amaranthaceae species had much lower TFC values, indicating that flavonoid levels vary on a large scale among the different samples.

### 3.2. Quantification of Antioxidant Capacity

**Figure 2:**
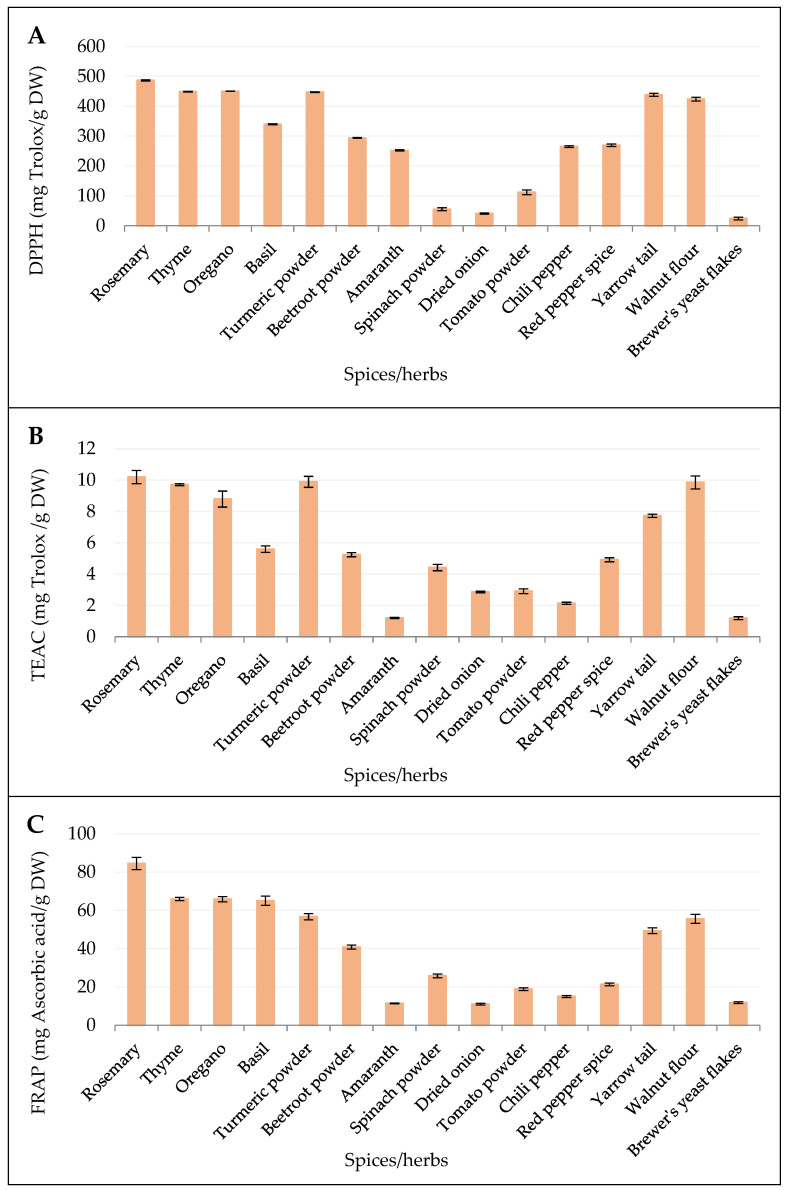
Antioxidant capacity in different plant species (spices and herbs) measured using different methods: (**A**) DPPH, (**B**) TEAC, and (**C**) FRAP techniques. Data are means ± SD.

The DPPH antioxidant capacity assays conducted for the 15 studied samples revealed significant variability across the different species, plant families, and tissues (Figure 2). Among the herbs, rosemary exhibited the highest antioxidant capacity (486.6 mg/g), followed by oregano (450.3 mg Trolox/g DW), thyme (449.1 mg Trolox/g DW), and turmeric (447.2 mg Trolox/g DW). These herbs, chiefly from the Lamiaceae and Zingiberaceae families, demonstrate strong antioxidant properties. Noteworthy is that antioxidant activity was also observed for walnut flour (423.9 mg Trolox/g DW) and yarrow tail (438.5 mg Trolox/g DW). In contrast, some samples, such as brewer’s yeast flakes (24.3 mg Trolox/g DW), dried onion (40.9 mg Trolox/g DW), and spinach powder (55.3 mg Trolox/g DW), presented much lower antioxidant activities, underscoring the variability in antioxidant capacity between different plant tissues and yeast. This highlights the more pronounced antioxidant capacity of herbs compared to other tissues like bulbs and leaves.

The antioxidant capacities established for the 15 studied samples using the TEAC method revealed notable alterations, as some species exhibited significantly higher values than the others. Rosemary demonstrated the highest antioxidant capacity (10.20 mg Trolox/g DW) among all of the samples, closely followed by turmeric powder (9.90 mg Trolox/g DW), walnut flour (9.86 mg Trolox/g DW), and thyme (9.72 mg Trolox/g DW). Other Lamiaceae species, such as oregano (8.79 mg Trolox/g DW) and basil (5.59 mg Trolox/g DW), also showed relatively high antioxidant capacities, though lower than those of rosemary and thyme. Among the members of Amaranthaceae species, beetroot (5.24 mg Trolox/g DW) and spinach powder (4.42 mg Trolox/g DW) displayed moderate antioxidant capacities, while amaranth exhibited one of the lowest values (1.20 mg Trolox/g DW). Solanaceae species, such as tomato powder (2.91 mg Trolox/g DW), chili pepper (2.13 mg Trolox/g DW), and red pepper spice (4.92 mg Trolox/g DW), exhibited lower antioxidant capacities compared to that of the members of the Lamiaceae family. Yarrow tail (Asteraceae) exhibited a moderate TEAC value of 7.72 mg Trolox/g DW, while brewer’s yeast flakes showed one of the lowest antioxidant capacities (1.19 mg Trolox/g DW). Overall, species from the Lamiaceae family displayed the highest antioxidant capacities, while Amaranthaceae and yeast samples showed lower values, highlighting the variability in antioxidant potential across different plant families and tissues.

The antioxidant capacity (reducing power) established using the FRAP method revealed significant variability across the 15 samples. Rosemary exhibited the highest FRAP antioxidant capacity (84.5 mg ascorbic acid/g DW), making it the most potent agent among the tested species. Other Lamiaceae species, including thyme (65.9 mg ascorbic acid/g DW), oregano (65.9 mg ascorbic acid/g DW), and basil (65.1 mg ascorbic acid/g DW), also demonstrated high reducing power. Turmeric powder, though stemming from a different family, showed a relatively strong antioxidant capacity (56.8 mg ascorbic acid/g DW), similar to walnut flour (55.6 mg ascorbic acid/g DW) from the Juglandaceae family. Yarrow tail (49.4 mg ascorbic acid/g DW) displayed a moderate antioxidant capacity compared to the other tested herbs and spices. Among the members of the Amaranthaceae family, beetroot powder (40.9 mg ascorbic acid/g DW) exhibited moderate antioxidant capacity, while amaranth (11.5 mg ascorbic acid/g DW) and spinach powder (25.8 mg ascorbic acid/g DW) displayed considerably lower values. Solanaceae species, such as tomato powder (18.9 mg ascorbic acid/g DW), chili pepper (15.1 mg ascorbic acid/g DW), and red pepper spice (21.4 mg ascorbic acid/g DW), showed lower reducing power compared to the Lamiaceae and other highly potent species. The lowest antioxidant capacities were observed for brewer’s yeast flakes (11.9 mg ascorbic acid/g DW) and dried onion (11.0 mg ascorbic acid/g DW). Overall, species from the Lamiaceae family consistently demonstrated the highest antioxidant capacities, while Amaranthaceae species and yeast samples exhibited much lower reducing power.

### 3.3. Correlation Between Total Polyphenol and Flavonoid Contents and DPPH, TEAC, and FRAP Values

The correlation analysis revealed significant relationships between total polyphenol content (TPC), total flavonoid content (TFC), and the antioxidant assays (DPPH, TEAC, and FRAP) (Table 3). However, it is important to note that these correlations are influenced by the unique antioxidant profiles of each plant species. For example, Lamiaceae species such as rosemary, thyme, and oregano, which are rich in phenolic acids (e.g., rosmarinic acid, caffeic acid) and flavonoids (e.g., apigenin, luteolin), exhibited strong correlations between TPC and antioxidant assays (r = 0.772–0.913). This is consistent with their high antioxidant capacities, as these compounds are known to effectively scavenge free radicals and reduce oxidative stress.

In contrast, Amaranthaceae species such as beetroot and spinach, which contain lower levels of polyphenols but are rich in other bioactive compounds like betalains and carotenoids, showed weaker correlations (r = 0.201–0.499). This suggests that the antioxidant activity in these species may be driven by mechanisms not fully captured by TPC or TFC measurements. Similarly, Solanaceae species such as tomato and chili pepper, which contain capsaicinoids and lycopene, displayed moderate correlations, indicating that their antioxidant profiles are influenced by a combination of polyphenols and other bioactive components.

These species-specific variations highlight the importance of considering the unique composition of antioxidant molecules in each plant when interpreting correlation results. While TPC and TFC are useful indicators of antioxidant potential, they may not fully capture the contributions of other bioactive compounds, such as carotenoids, tocopherols, or alkaloids, which can also play significant roles in antioxidant activity. The finding that the used antioxidant assays (DPPH, TEAC, FRAP) consistently show strong correlation with TPC has already been verified in the literature [60,61,62]. However, the antioxidant profiles of plants may exhibit such a large variation that it makes it impossible to make unambiguous estimations on the correlation between antioxidant assays and the TPC values. Thus the involvement of various antioxidant assays and several plant species is necessary to obtain authentic and well-grounded conclusions on the issue. The aim of our studies was not to determine the profile of phenolic compounds, but to compare the antioxidant capacities in plants and to establish plausible correlations.

Regression analysis underscores the significant alignment among these assays (Table 4). In particular, TEAC and FRAP display a high coefficient of determination (R^2^ = 0.8061), indicating that over 80% of the variations in FRAP values might also be experienced by parallel TEAC measurements. This result suggests that these two assays are not only highly correlated, but also have similar predictive abilities regarding antioxidant activity in cases of the studied samples, making them effective, interchangeable methods that reinforce one another. Additionally, DPPH and FRAP display a moderately strong regression relationship (R^2^ = 0.689), where nearly 69% of the variations in FRAP values might be explained by DPPH, highlighting the prospects of DPPH to provide reliable predictions for results gained by FRAP assay. TEAC and DPPH also exhibit a strong regression relationship (R^2^ = 0.6802), reinforcing the notion that these two methods capture overlapping antioxidant properties, though unique insights are gained by regarding their operational differences.

All in all, these findings reveal that while DPPH, TEAC, and FRAP are capable of featuring antioxidant capacity, they share a substantial degree of sensitivity to similar compounds, especially polyphenols, in the spices and herbs tested. The high correlations among these assays suggest that they can reliably reflect antioxidant activity across diverse plant-based samples, with FRAP demonstrating slightly stronger predictive associations with TEAC and DPPH. This close alignment may indicate that the implied assays, particularly FRAP and TEAC, are considered robust tools for assessing antioxidant properties in polyphenol-rich foods. These results provide a deeper understanding of the comparability, applicability, and susceptibility of these antioxidant assays, indicating their prospective utilization not only in the case of isolated measurements, but also as part of comprehensive antioxidant assessment toolkits.

## 4. Discussion

### 4.1. The Role of TPC and TFC in Antioxidant Capacity

In this study, TPC and TFC were found to be significant parameters to feature antioxidant capacity in selected herbs and spices, with strong correlations particularly evident in the DPPH and TEAC assays. High TPC values proved to be the most notable in species from the Lamiaceae family, such as rosemary, thyme, and oregano, which demonstrated superior antioxidant capacities across all three assays. These findings support previous research by Yashin et al. [23], which emphasized polyphenols’ pivotal role in scavenging free radicals, particularly through the DPPH assay, due to their molecular structure that facilitates single-electron transfer [63]. In their study, they identified Lamiaceae species as plants rich in polyphenols, endowing them with robust antioxidant capacities in both hydrophilic and lipophilic environments [64]. Several mechanisms contribute to the relevance of polyphenols in antioxidant assays, as polyphenols act as radical scavengers by donating hydrogen atoms, transferring electrons, or through metal chelation [65]. Consistent with Bhuyan and Handique’s findings, our study identified a correlation coefficient of r = 0.772 between TPC and DPPH, further supported by the structural characteristics of polyphenols that facilitate their radical-scavenging properties, specifically in assays sensitive to single-electron transfer (SET) mechanisms like DPPH [66]. Notably, species like rosemary and thyme, which exhibited high TPC levels, were consistently ranked among those with the highest antioxidant capacity, confirmed by DPPH and TEAC assays. These results support the assertion that polyphenols in Lamiaceae herbs have a high antioxidant potential due to their molecular structure, which promotes both electron donation and radical stabilization.

### 4.2. Comparative Analysis of TPC and Antioxidant Capacity in TEAC, DPPH, and FRAP Assays

The significant correlation (r = 0.856) between TPC and TEAC certified in our findings suggests that TPC may be a highly consistent indicator of antioxidant potential in TEAC assays, by means of its capability of detection of both hydrophilic and lipophilic antioxidant agents. This correlation aligns with the results reported by Rumpf et al. [67], who documented that polyphenol compounds of various herbs showed enhanced TEAC values, particularly in cases of species with higher phenolic acid content [44]. Rumpf’s findings highlight the potential of TEAC assays to capture a broader spectrum of antioxidant activity compared to DPPH and FRAP, as the ABTS radical used in TEAC can interact with a diverse range of antioxidant compounds, including both hydrophilic and hydrophobic molecules. The strong correlation observed between TPC and FRAP (r = 0.913) in our study suggests that polyphenols are substantial contributors to the reducing power, reflecting their impact on FRAP-measured antioxidant activity. This pattern is in accordance with analyses by Yu et al. [68], where the reducing power established by FRAP varied significantly across plant extracts with similar TPC, suggesting that FRAP is influenced by specific polyphenol subclasses or other bioactive components. In our study, this trend was evident for Lamiaceae species like oregano, which displayed high TEAC and DPPH but moderate FRAP activity, suggesting that its polyphenol profile may be less effective in terms of reducing power compared to other species like rosemary.

### 4.3. Correlation Between TFC and Implied Antioxidant Assays

Flavonoids, a subgroup of polyphenols, are particularly efficient in scavenging ROS, and are known to enhance antioxidant capacity, especially in DPPH and TEAC assays. In our study, TFC demonstrated a moderate correlation with DPPH (r = 0.425) and TEAC (r = 0.499), aligning with the prior findings of Chen et al. [69], who observed that a high flavonoid content in botanical extracts from medicinal plants significantly boosted the DPPH radical scavenging activity. This correlation underscores the efficiency of flavonoids in neutralizing radicals through hydrogen atom donation, a mechanism particularly effective in DPPH assays, which respond well to flavonoids’ antioxidant properties. Furthermore, the significant TFC-TEAC correlation suggests that flavonoids contribute to the overall antioxidant potential, as indicated by high TEAC values for species like turmeric and thyme. This outcome corroborates reports by Sharma and Singh [59], who emphasized the role of flavonoids in TEAC antioxidant activity, especially in lipophilic systems where flavonoids can interact with hydrophobic radicals. The low correlation between TFC and FRAP (r = 0.355) pointed out in our study, while supportive of flavonoids’ impact, indicates that FRAP may be less sensitive to flavonoids compared to polyphenols, as the reducing power of flavonoids may vary based on structural characteristics and molecular weight [70].

### 4.4. Comparative Antioxidant Profiles Among Plant Families

The antioxidant capacities of the studied plant families varied significantly, reflecting differences in their bioactive compound profiles. Lamiaceae species, such as rosemary and thyme, consistently demonstrated high TPC and TFC values, along with superior antioxidant capacities across all assays. This is attributed to their rich content of phenolic acids and flavonoids, which are highly effective in radical scavenging and electron transfer mechanisms.

In contrast, Amaranthaceae species, including beetroot and spinach, exhibited lower TPC and TFC values, as well as reduced antioxidant activities. This may be due to their reliance on other bioactive compounds, such as betalains in beetroot, which contribute to antioxidant activity through mechanisms not fully captured by TPC or TFC measurements. Similarly, Solanaceae species such as tomato and chili pepper, which contain capsaicinoids and carotenoids, displayed moderate antioxidant capacities, reflecting the combined contributions of polyphenols and other bioactive components.

These findings underscore the importance of considering species-specific antioxidant profiles when interpreting correlation results. While TPC and TFC provide valuable insights into polyphenol-driven antioxidant activity, they may not fully account for the contributions of other bioactive compounds, which can vary significantly across plant families and species.

By comparing the antioxidant capacities among plant families, our study highlighted a clear distinction between the Lamiaceae and the Amaranthaceae species. Herbs from the Lamiaceae family, such as rosemary and thyme, consistently demonstrated high TPC and TFC values, coupled with superior antioxidant capacities confirmed by DPPH, TEAC, and FRAP assays. This observation aligns with the findings of Sik et al. [71], who reported high polyphenol contents and strong antioxidant properties among Lamiaceae herbs due to the presence of rosmarinic acid and other phenolic acids. The antioxidant richness of these herbs underscores their potential use as dietary supplements for enhancing antioxidant intake and mitigating oxidative-stress-related damages. Amaranthaceae species, including beetroot and spinach, demonstrated comparatively lower TPC and TFC values, as well as antioxidant activities. Previous studies, such as those by Bang et al. [72], similarly documented low to moderate antioxidant capacities in Amaranthaceae, attributing this to the lower concentration of polyphenols and flavonoids compared to the Lamiaceae species. Our presented results reinforce the idea that antioxidant activity in Amaranthaceae species may stem from other bioactive compounds like betalains in beetroot, which contribute to a moderate antioxidant activity, but are less effective in scavenging free radicals compared to the phenolic-rich herbs.

### 4.5. Implications of Antioxidant Assays on Compound Specificity

The distinct correlations observed in our study between diverse antioxidant assays and TPC as well as TFC levels underscore the unique response exhibited by each assay to the specific antioxidant compounds. The DPPH and TEAC assays’ moderate correlation with TFC values indicates their sensitivity to flavonoids, which excel in radical scavenging due to their hydroxyl group configurations [73]. TEAC’s ability to detect both hydrophilic and lipophilic antioxidants makes it a versatile assay, being especially suitable for the examination of flavonoid-rich extracts, which may explain the high TEAC readings for turmeric and thyme in our study. On the other hand, FRAP, which measures reducing power, showed a strong correlation with TPC (r = 0.913), suggesting that the assay may chiefly be influenced by polyphenol compounds. This was also noted by Mitrović et al. [74], who reported that FRAP values do not always correlate with polyphenol content due to the assay’s dependence on the sample’s specific redox potential. In cases of species like walnut, which showed moderate FRAP but high TPC and TEAC values, the data suggest that polyphenols may not be the primary contributors to reducing power, which could also be largely influenced by specific amino acids or carotenoids present in walnuts.

### 4.6. Correlation Analysis and Statistical Insights

Our study’s correlation analysis revealed a strong positive correlation between FRAP and DPPH assays (r = 0.844), and a similarly high correlation between TEAC and FRAP assays (r = 0.907), indicating consistent results across these assays. Such correlations suggest overlapping mechanisms among the assays, particularly for species with robust antioxidant profiles like rosemary and thyme. These findings mirror conclusions from studies by Matkovits et al. [75], where high inter-assay correlations were similarly observed among extracts with high phenolic and flavonoid content. However, the slightly lower FRAP-TEAC correlation (r = 0.907) in our study may reflect variability in the response of different compounds to the assays, as FRAP tends to underrepresent non-polyphenol antioxidants.

The correlation analysis of antioxidant activity assays (FRAP, DPPH, and TEAC) reveals significant insights into their relationships and applicability. Among the assays, FRAP and TEAC exhibit the highest inter-assay correlation (r = 0.907), indicating a strong alignment in their ability to measure antioxidant activity, likely due to their sensitivity to similar polyphenol-driven mechanisms. DPPH also demonstrates high correlations with both FRAP (r = 0.844) and TEAC (r = 0.845), confirming its reliability as an antioxidant measure despite methodological differences.

When evaluating the relationship between TPC and the assays, FRAP emerges as the strongest indicator (r = 0.913), followed by TEAC (r = 0.856) and DPPH (r = 0.772). This suggests that polyphenols are key contributors to the antioxidant capacities measured by these methods. In contrast, TFC shows weaker correlations with TEAC (r = 0.499) and DPPH (r = 0.425) demonstrating moderate associations, underscoring the lesser contribution of flavonoids compared to polyphenols in these assays.

The FRAP-TEAC correlation, however, indicates that certain compounds may preferentially contribute to either the reducing power or the radical scavenging ability. Species like walnut, which displayed moderate FRAP but high DPPH and TEAC values, suggest that antioxidant capacity is not solely dependent on polyphenols or flavonoids but may also be influenced by other bioactive compounds such as tocopherols or carotenoids. This highlights the importance of a multi-assay approach in accurate featuring of the antioxidant potential of diverse plant species, as each assay may reflect different aspects of the antioxidant action.

### 4.7. Implications for Future Research and Nutritional Applications

The results from this study underscore the importance of polyphenols and flavonoids as primary contributors to antioxidant capacity, particularly in herbs and spices with high TPC and TFC. The strong correlation between TPC and TFC with values obtained using DPPH and TEAC assays indicates that these compounds are regarded as a key to radical scavenging potential as well as electron-transfer-induced antioxidant capacity, which has important implications for dietary and therapeutic practices. Lamiaceae herbs, such as rosemary and thyme, emerged as potent sources of antioxidants that can be incorporated into dietary regimens to combat oxidative-stress-related conditions. Additionally, our findings suggest that TPC and TFC are reliable indicators of antioxidant capacity, which could be integrated into plant growing programs focusing on enhancing polyphenol and flavonoid content in specific plant families. This study also suggests that antioxidant research would benefit from a multi-assay approach to fully capture the spectrum of antioxidant activity. Each assay’s unique sensitivity to polyphenols, flavonoids, or other bioactive components reveals a complex interplay of compounds contributing to antioxidant capacity. By employing our results, future studies can characterize the antioxidant profiles of underexplored plant species in an improved manner, and might gain deeper insight into the relationship between bioactive compounds and health implications.

## 5. Conclusions

This study demonstrated that the FRAP assay exhibited the strongest correlation with total polyphenol content (TPC) (r = 0.913), confirming its effectiveness in measuring reducing power, particularly in polyphenol-rich samples. The TEAC assay showed a strong correlation with TPC (r = 0.856) and proved versatile in detecting both hydrophilic and lipophilic antioxidants, making it a valuable method for assessing antioxidant capacity. While the DPPH assay was slightly less correlated with TPC (r = 0.772), it remained reliable for evaluating radical scavenging activity, especially in samples with high polyphenol content. Species from the Lamiaceae family, such as rosemary, thyme, and oregano, consistently displayed high antioxidant activities across all assays, highlighting their rich polyphenol and flavonoid profiles. Significant variability in antioxidant capacity was observed among plant families, with Lamiaceae species showing the highest activities and Amaranthaceae species exhibiting lower values, likely due to differences in bioactive compound composition. The high inter-correlation between the FRAP, TEAC, and DPPH assays (r = 0.844–0.907) underscores their complementary nature in capturing different aspects of antioxidant activity, emphasizing the importance of a multi-assay approach for a comprehensive evaluation of antioxidant potential. However, the species-specific variations in antioxidant profiles highlight the need to consider the unique composition of bioactive compounds in each plant when interpreting correlation results.

## Figures and Tables

**Table 1:** Classification, antioxidant compounds, and biological importance of selected spices, herbs, and yeast.

Name	Family	Classification	Major Antioxidant Components	Important Biological Activities	Reference
Rosemary (*Salvia rosmarinus* L.)	Lamiaceae	herb	Phenolics: caffeic acid, rosmarinic acid carnosic acid, carnosol, and hesperidin Flavonoids: diosmin	Antioxidant, anti-inflammatory, antimicrobial, antiviral, anti-metabolic syndrome, anticarcinogenic, antimutagenic, antinociceptive, neuroprotective	[22]
Thyme (*Thymus vulgaris* L.)	Lamiaceae	herb	Phenolics: thymol and carvacrol Flavonoids: apigenin	Antioxidant, antimicrobial, expectorant, spasmolytic, mucolytic, antitussive	[24,25]
Oregano (*Origanum vulgare* L.)	Lamiaceae	herb	Phenolics: rosmarinic, chlorogenic, cinnamic, caffeic, syringic, benzoic, vanillic, gallic, chicoric, and 2,4-dihydroxybenzoic acids Flavonoids: quercetin, apigenin, luteolin, naringenin, and kaempferol	Antioxidant, antifungal, antimicrobial, expectorant, stimulant, carminative, anticancer, antiaging agent	[26]
Basil (*Ocimum basilicum* L.)	Lamiaceae	herb	Phenolics: rosmarinic acid, caffeic acid, chicoric acid, and ferulic acid Flavonoids: apigenin, luteolin, and quercetin	Antioxidant, antibacterial, antifungal, anti-inflammatory, antidiabetic	[27,28]
Turmeric powder (*Curcuma longa* L.)	Zingiberaceae	spice	Phenolics: curcumin and turmerones Flavonoids: quercetin, luteolin, and rutin	Antioxidant, antimicrobial, anti-inflammatory, anticancer, hypoglycemic, anticoagulant	[27]
Beetroot powder (*Beta vulgaris* L.)	Amaranthaceae	spice	Phenolics: chlorogenic acid, caffeic acid, and catechin Flavonoids: betagarin, betavulgarin, quercetin, and rutin Carotenoids	Antioxidant, antiviral, antibacterial, antianemic, anti-inflammatory, antihypertensive	[29,30]
Amaranth (*Amaranthus* spp.)	Amaranthaceae	pseudo-cereal	Phenolics: caffeic acid and ferulic acid Flavonoids: rutin and quercetin	Antioxidant, antimicrobial, anti-inflammatory, antimalarial, antidiabetic, anticarcinogenic, hepatoprotective	[11]
Spinach powder (*Spinacia oleracea* L.)	Amaranthaceae	spice	Phenolics: coumaric acid, ferulic acid, and caffeic acid Flavonoids: patuletin, spinacetin, quercetin, and luteolin Carotenoids	Antioxidant, antimicrobial, anticancer,	[31]
Dried onion (*Allium cepa* L.)	Amaryllidaceae	spice	Phenolics: phenolic acids and anthocyanins Flavonoids: quercetin, rutin, and kaempferol	Antioxidant, anti-inflammatory, immunomodulatory, anticancer	[32,33]
Tomato powder (*Solanum lycopersicum* L.)	Solanaceae	spice	Phenolics: chlorogenic acid, caffeic acid, and vanillic acid Flavonoids: rutin, quercetin, and kaempferol Carotenoids	Antioxidant, anticarcinogenic, cardioprotective, antimicrobial, anti-inflammatory	[34]
Chili pepper (*Capsicum annuum* L.)	Solanaceae	spice	Flavonoids: apigenin, luteolin, and quercetin Carotenoids Capsaicinoids: capsaicin	Antioxidant, anti-inflammatory, antimicrobial, anti-obesity	[35,36]
Red pepper (*Capsicum annuum* L.)	Solanaceae	spice	Phenolics: gallic acid, vanillic acid, caffeic acid, coumaric acid, and chlorogenic acid Flavonoids: myricetin, quercetin, luteolin, and apigenin Capsaicinoids: capsaicin	Antioxidant, immunomodulatory, anticancer, antimutagenic, antiplatelet, antiangiogenic, anti-inflammatory, antiviral	[56]
Yarrow tail (*Achillea millefolium* L.)	Asteraceae	herb	Phenolics: dicaffeoylquinic acid Flavonoids: apigenin, luteolin, and quercetin	Antioxidant, anti-inflammatory, antiaging, antibacterial, antitumor, antidiabetic	[37,38]
Walnut flour (*Juglans regia* L.)	Juglandaceae	flour	Phenolics: chlorogenic acid and ferulic acid) Flavonoids: catechin and rutin Carotenoids	Antioxidant, anti-inflammatory, antibacterial, anticancer, neuroprotective effects, cholesterol-lowering activity	[39]
Brewer’s yeast flakes (*Saccharomyces cerevisiae*)	Saccharomycetaceae	yeast	Phenolics: gallic acid, protocatechuic acid, catechin, and ferulic acid B-vitamins: biotin and folic acid	Antioxidant, anti-inflammatory, antitumorigenic	[40,41]

**Table 2:** The source and form of spices/herbs examined in the current study.

Sample	Origin of Spice	Plant Part Used to Make Spice	Form of Spice Used to Make Extraction
Rosemary	Hungary	leaves	dry leaves
Thyme	Hungary	leaves	dry leaves
Oregano	Hungary	leaves	dry leaves
Basil	Hungary	leaves	dry leaves
Turmeric	India *	rhizomes	powder
Beetroot	Hungary	taproot	powder
Amaranth	Hungary	seeds	powder
Spinach	Hungary	leaves	powder
Dried onion	India *	bulbs	granulates
Tomato	Hungary	fruits	powder
Chili pepper	Hungary	placenta	flakes
Red pepper	Hungary	fruits	powder
Yarrow tail	Hungary	leaves	dry leaves
Walnut	Ukraine *	kernel	flour
Brewer’s yeast	Hungary	one cell fungus	flakes

* All spices/herbs were bought from a Hungarian store.

**Table 3:** Pearson correlation between total polyphenol (TPC) and flavonoid contents (TFC) and different methods of antioxidant activity quantification in 15 spices and herbs.

	TPC	TFC	DPPH	TEAC
TPC	1			
TFC	0.201	1		
DPPH	0.772 **	0.425	1	
TEAC	0.856 **	0.499	0.845 **	1
FRAP	0.913 **	0.355	0.844 **	0.907 **

** Correlation is significant at the 0.01 level (2-tailed).

**Table 4:** Regression (R^2^) between total polyphenol (TPC) and flavonoid contents (TFC) and different methods of antioxidant activity quantification in 15 spices and herbs.

	TPC	TFC	DPPH	TEAC
TFC	0.0405			
DPPH	0.5686	0.1951		
TEAC	0.7196	0.2777	0.6802	
FRAP	0.7808	0.1559	0.689	0.8061

## Data Availability

Data are included in the manuscript.
